# The Last Call

**Published:** 1904-08

**Authors:** 


### "THE LAST CALL."

To those familiar with vestibuled trains and long distance travel this ominous sound will be recalled as the last warning to belated feeders due in the dining-car. The writer has, however, no gastronomic idea connected with the title to this article. It belongs to a more important issue,—the last call to the hosts of dentistry in America to be stirring themselves to meet our colleagues from distant lands in St. Louis and, in Congress assembled, devise ways and means to make a standard of excellence for the profession of dentistry.

There is always an uncertain number who delay deciding whether to take part in a great movement or let the opportunity slip by forever. The close of this month will find three important conventions assembling at St. Louis,—the International Dental Federation, the International Dental Congress, and the National Dental Association. Before the September issue of this journal reaches our subscribers these great meetings will have concluded their labors.

It is supposed that it is scarcely necessary to remind our intelligent circle of readers that they have a duty to perform in this connection, and that duty lies not in the direction of an exposition, great as that undoubtedly is, but in a concentrated effort, shoulder to shoulder, to make such a representation of American dentistry that, in numbers, at least, the occasion may be made impressive. We would not be misunderstood here, for while numbers carry force, they cannot bear, in any convocation, a close relation to quality. The mob in science is to be deprecated as much as the mob in misrule. Dentistry, however, should not be a mob, in the ordinary acceptation of that term. The time has arrived when all who claim the title of stomatologist, and all dentists do so claim, have received a training that warrants the expectation that they are all drilled soldiers in the army of dental science and will fall into the ranks as naturally as the German or French reserve will enter into active service with his regiment.

The doubtful man will argue with himself, "Of what use will I be there among the thousands? Every one cannot prepare papers of scientific value, and for myself, as I cannot write or speak, it will be time and money wasted for me to be present." This, in effect, is the personal argument with many. It is the self-depreciation that multiplies the dead weight in all the active work of the world. Every man, in the generic sense, has a work to do, and to shirk it without good reason is but little short of a crime. The common thief takes that which he has never earned. The man who is willing to accept an improvement that advances his profession without returning an equivalent is simply absorbing that which does not by right belong to him. While it is true that all are not equally gifted, and some cannot, or, perhaps, think they cannot, aid in organized effort, no one is so incapable as not to be able to give the light of his countenance to the labor, and thus encourage others to do the work he professes his inability to accomplish.

There is another and, possibly, a selfish side to this subject. The man or woman interested in dentistry cannot afford to miss any of the conventions, and especially those that will meet at St. Louis. He or she who feels that all the good of these meetings can be gathered from the journal reports lives in a "fool's paradise." The visual, the objective, is the training of value, and that many never avail themselves of this is evidenced all around us in a practical inability to become masterful in their calling.

Now, therefore, is it the last call to you, wherever you may be, to send in your membership fee. It means ten dollars of personal responsibility. You are, to that extent, a stockholder in the last great convocation that will meet in many years on this side of the Atlantic. The present generation will have become grizzled with age before the world of dentistry will again gather in our country. In all human probability the last of the great expositions in America will have been held when that at St. Louis closes its doors. The cost, which has gradually grown to such enormous figures, precludes the possibility of another of similar proportions. *Then grasp the present opportunity.*

From indications the membership list will exceed anything heretofore known of international dental congresses. This one promises to be an exposition in itself, for our dental manufacturers are making every effort to give an exhibit worthy the occasion. Dentistry in America will be on trial. Whether we have anything new to show or not may not be so important, but let the best be shown, and whether it be on the scientific or the practical side, may that be worthy of our people. Therefore, let every practitioner heed the last call to duty, and during this month, August, find the ways and means to reach St. Louis and to then actively enter into unison with the host that will gather there from all the civilized centres of the world.

